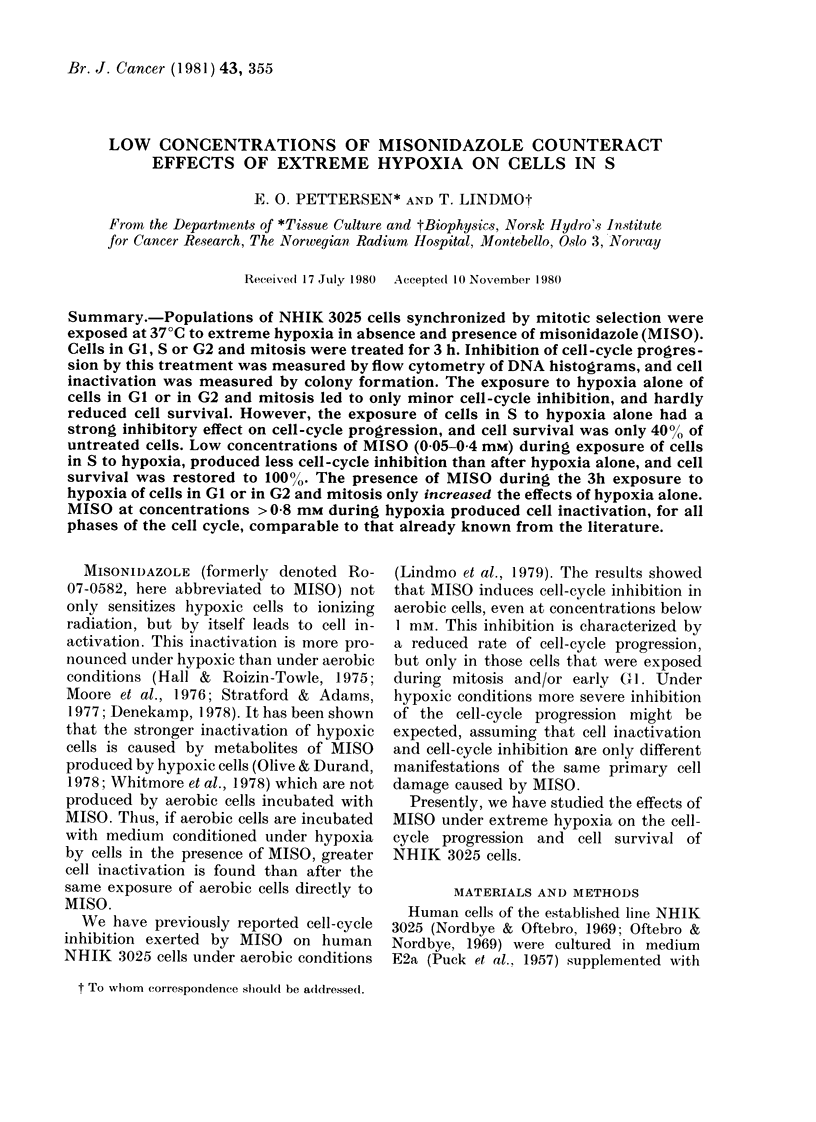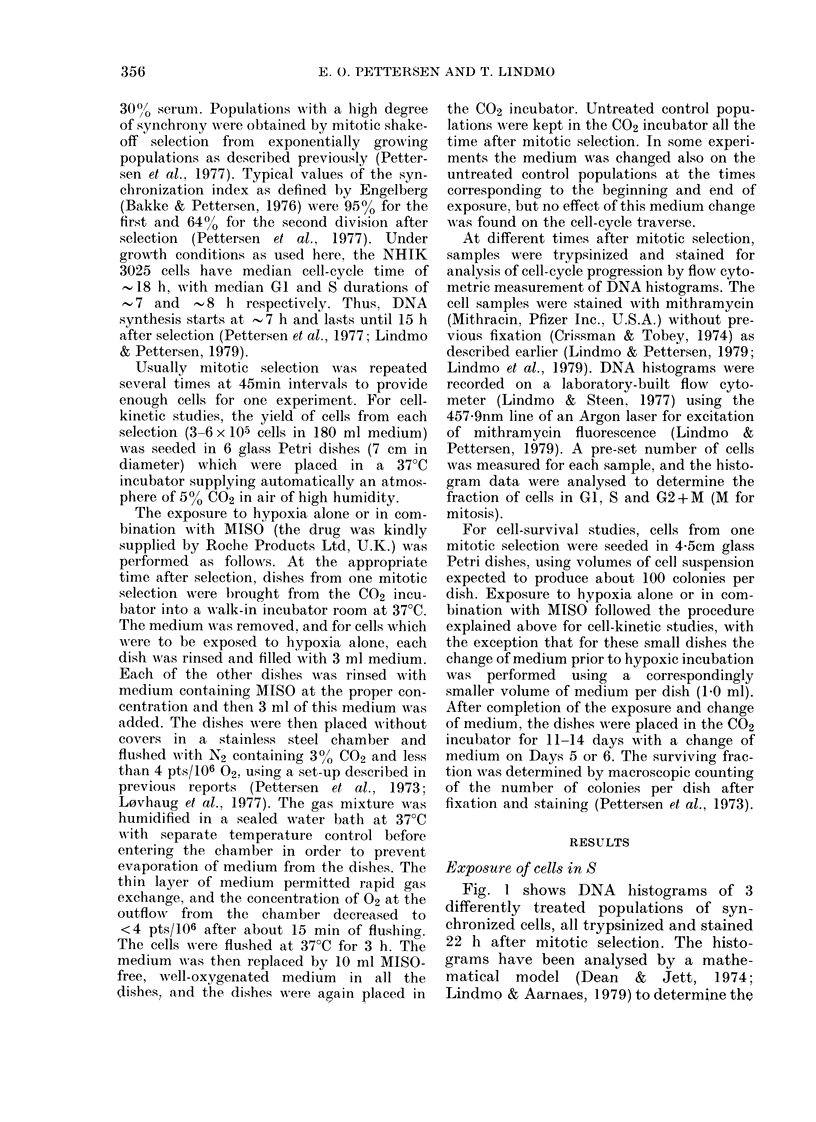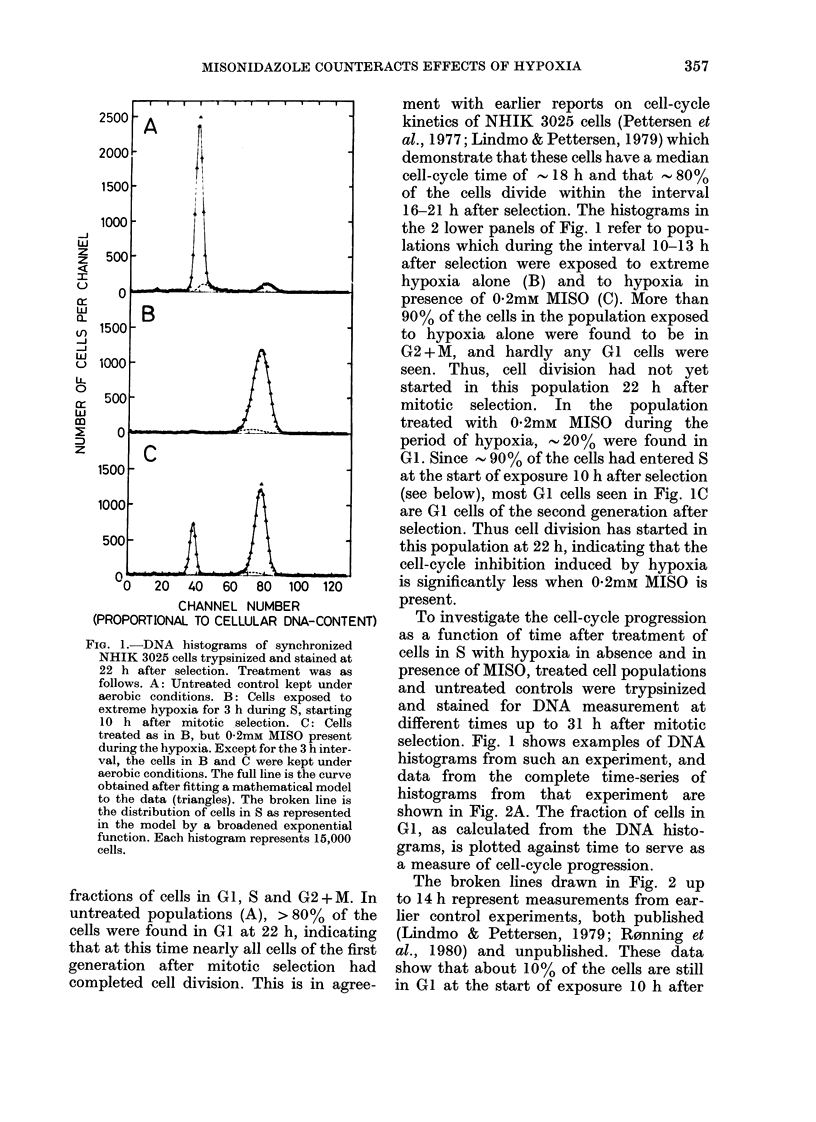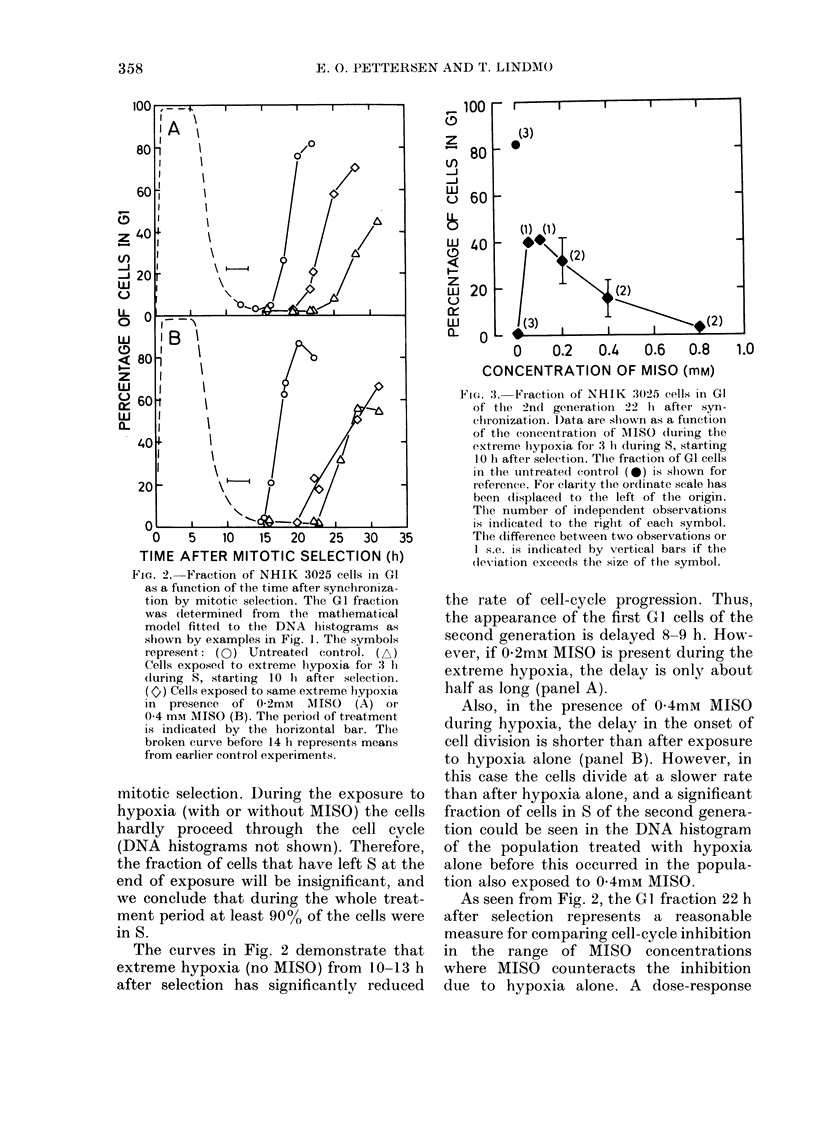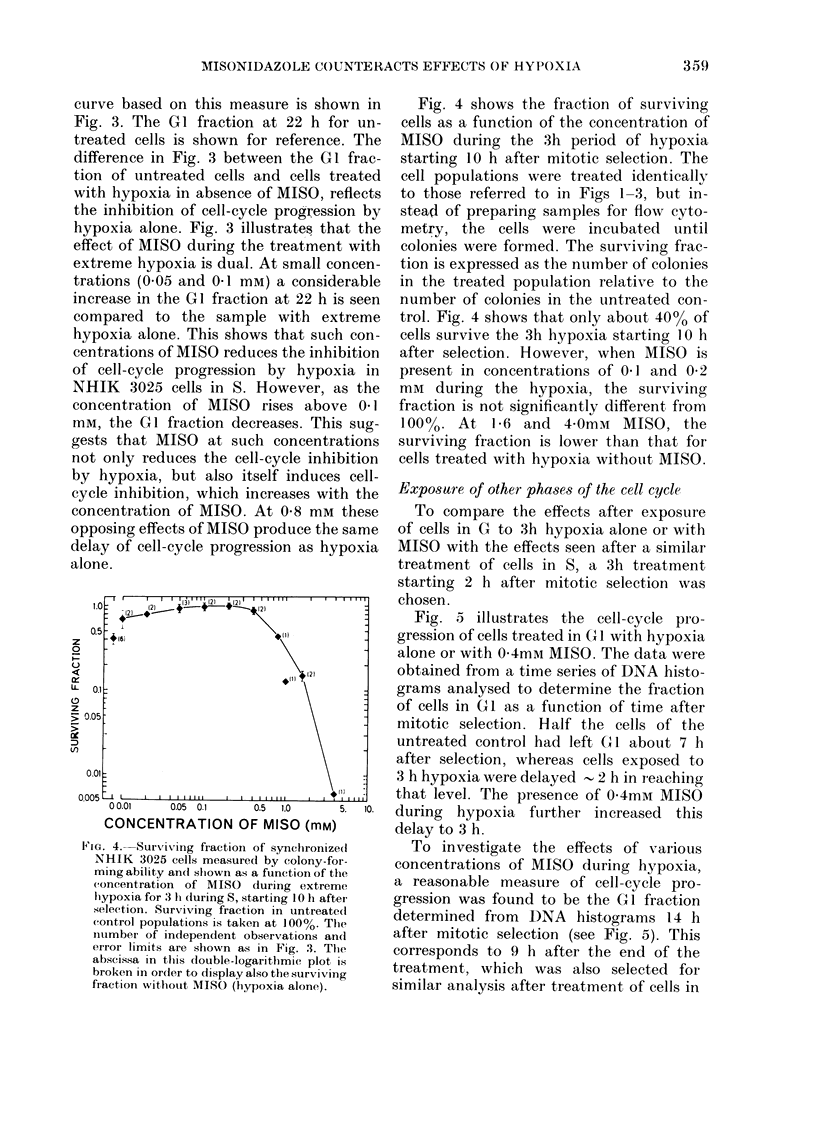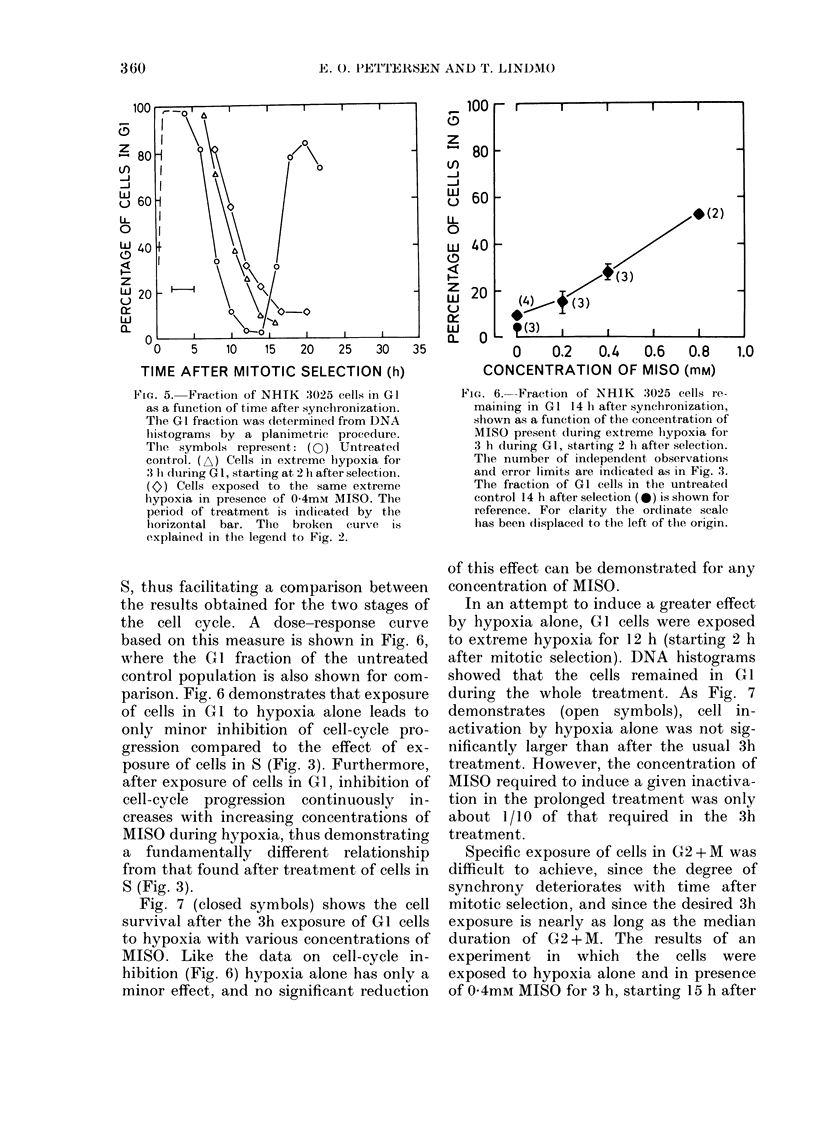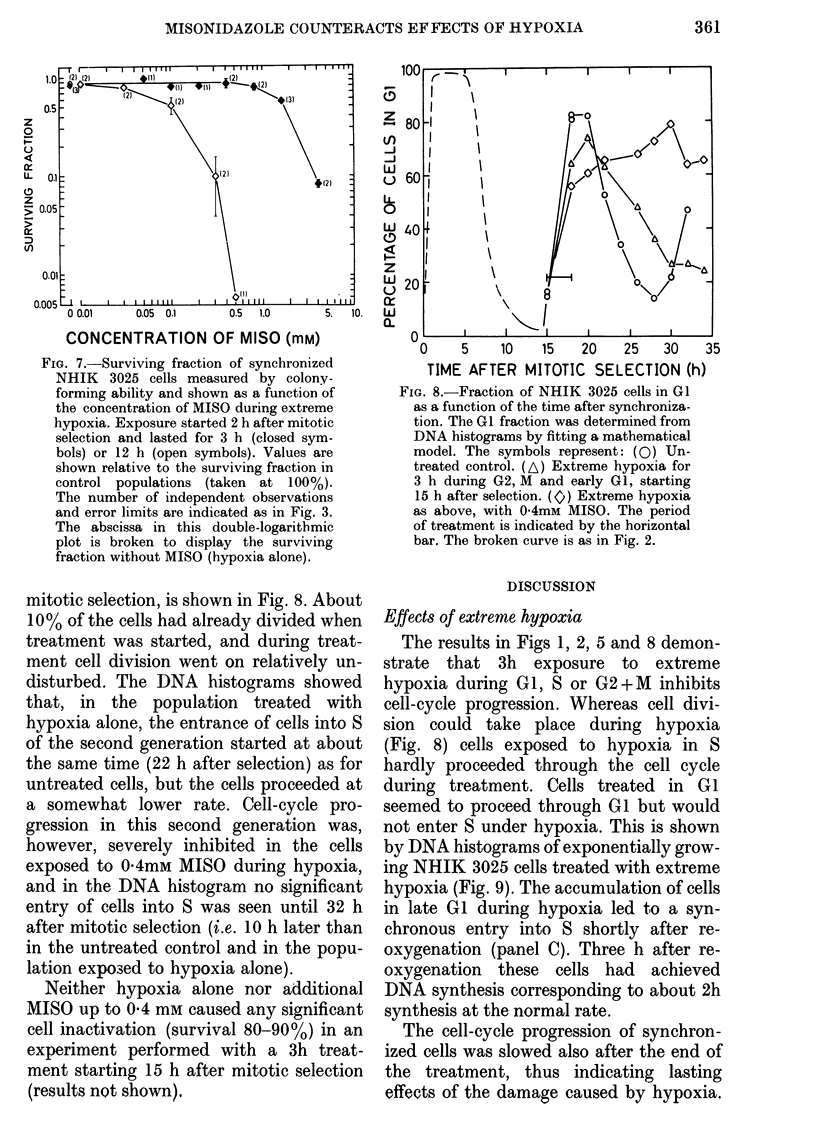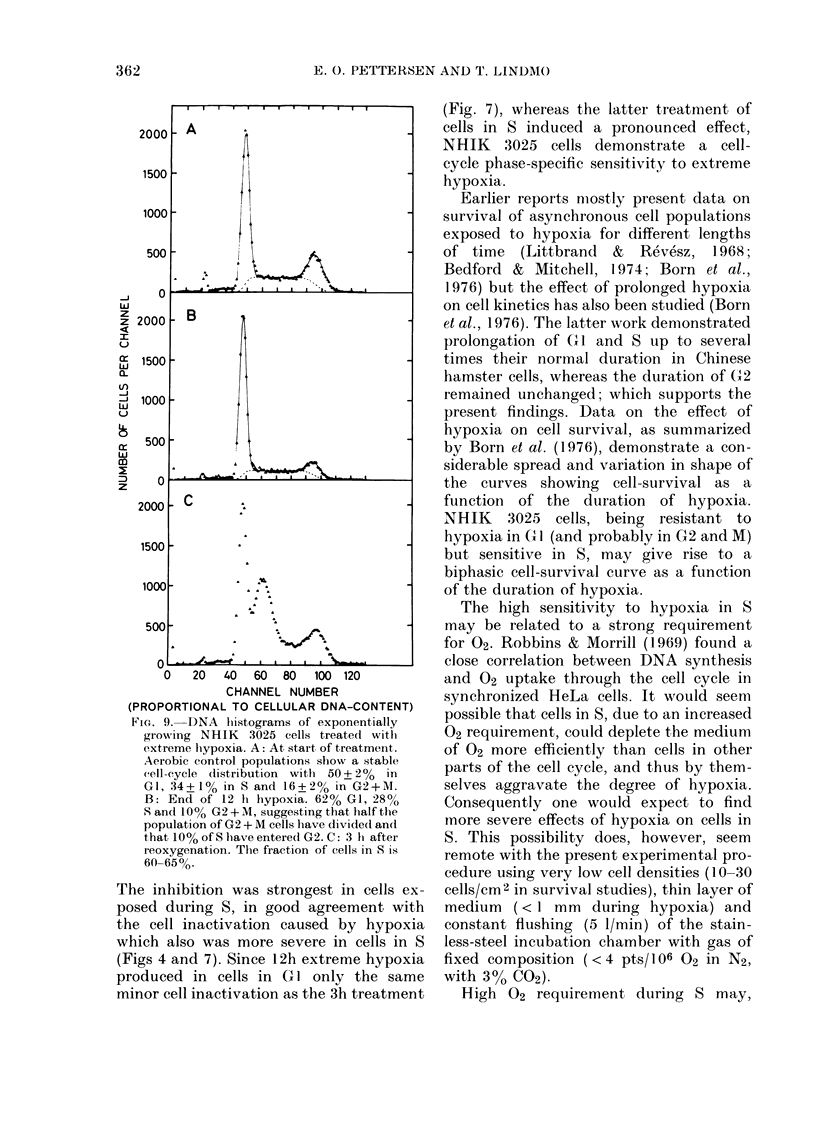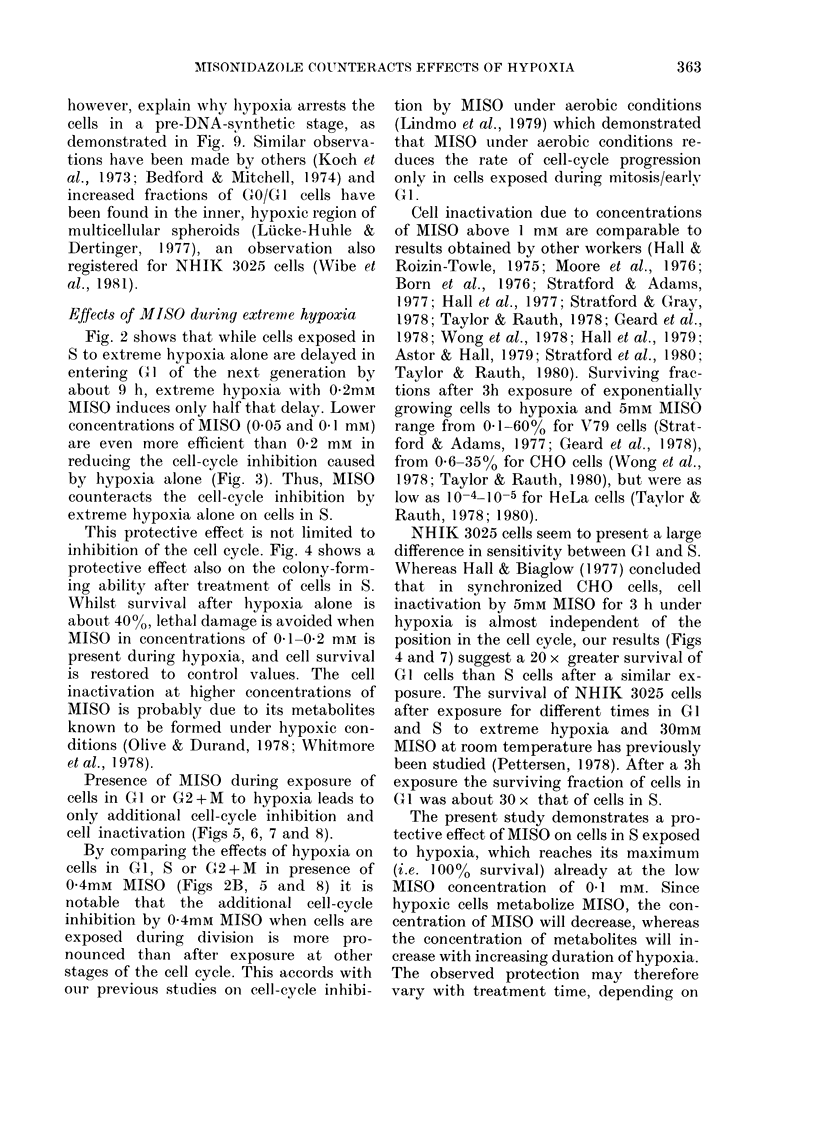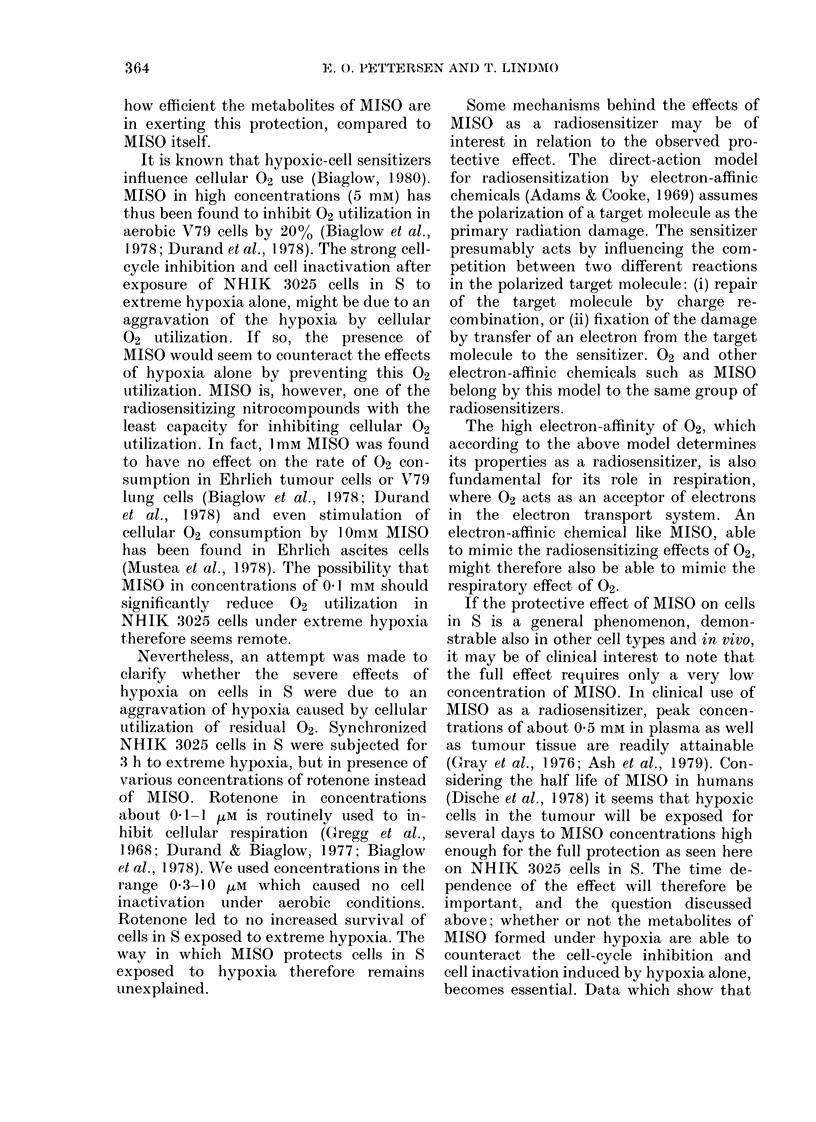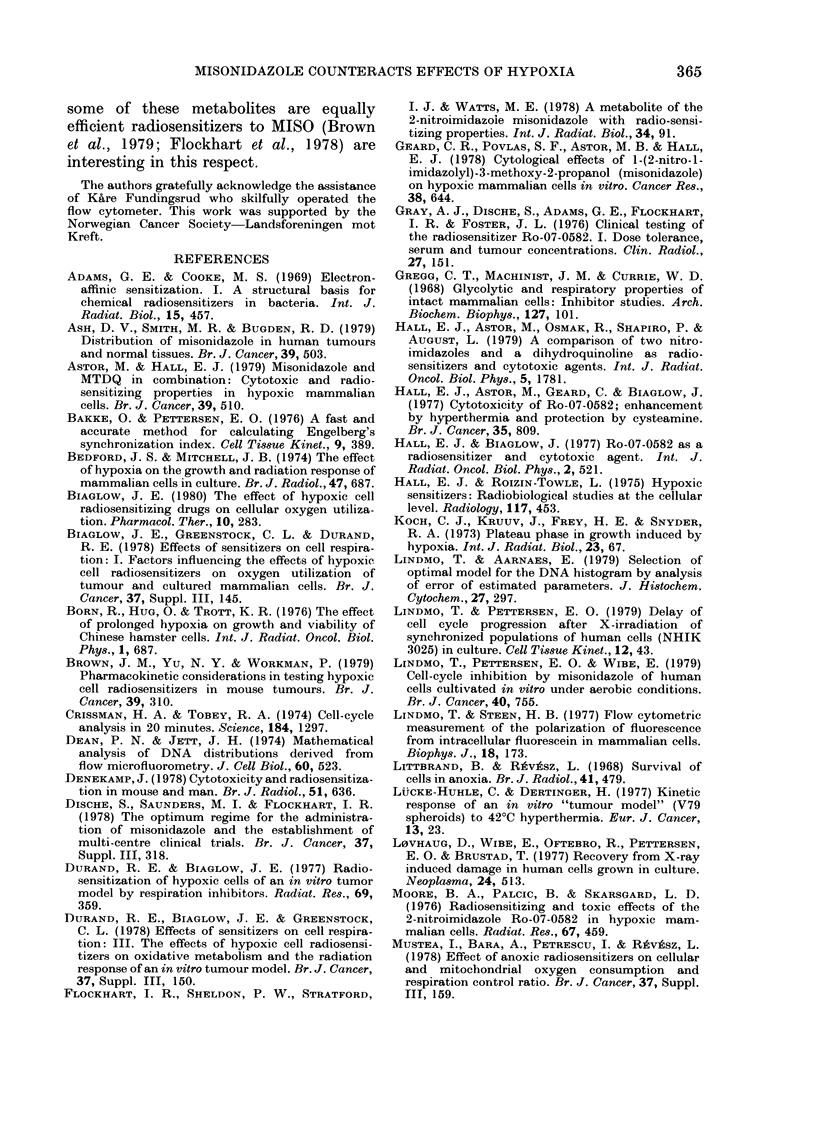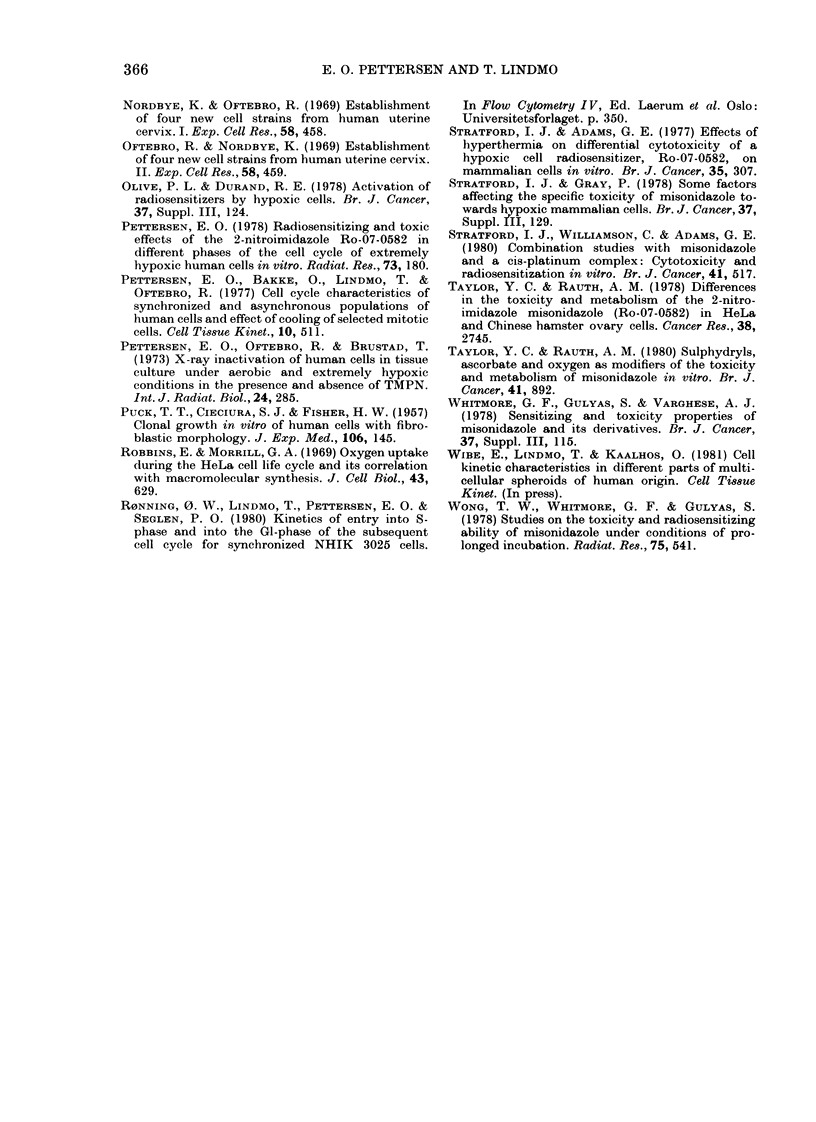# Low concentrations of misonidazole counteract effects of extreme hypoxia on cells in S.

**DOI:** 10.1038/bjc.1981.56

**Published:** 1981-03

**Authors:** E. O. Pettersen, T. Lindmo

## Abstract

Populations of NHIK 3025 cells synchronized by mitotic selection were exposed at 37 degrees C to extreme hypoxia in absence and presence of misonidazole (MISO). Cells in G1, S or G2 and mitosis were treated for 3 h. Inhibition of cell-cycle progression by this treatment was measured by flow cytometry of DNA histograms and cell inactivation was measured by colony formation. The exposure to hypoxia alone of cells in G1 or in G2 and mitosis led to only minor cell-cycle inhibition, and hardly reduced cell survival. However, the exposure of cells in S to hypoxia alone had a strong inhibitory effect on cell-cycle progression, and cell survival was only 40% of untreated cells. Low concentrations of MISO (0.05-0.4 mM) during exposure of cells in S to hypoxia, produced less cell-cycle inhibition than after hypoxia alone, and cell survival was restored to 100%. The presence of MISO during the 3h exposure to hypoxia of cells in G1 or in G2 and mitosis only increased the effects of hypoxia alone. MISO at concentrations greater than 0.8 mM during hypoxia produced cell inactivation, for all phases of the cell cycle, comparable to that already known from the literature.


*Br. J. Cancer* (1981) **43**, 355

### LOW CONCENTRATIONS OF MISONIDAZOLE COUNTERACT EFFECTS OF EXTREME HYPOXIA ON CELLS IN S

E. O. PETTERSEN* AND T. LINDMO†

*From the Departments of *Tissue Culture and †Biophysics, Norsk Hydro's Institute for Cancer Research, The Norwegian Radium Hospital, Montebello, Oslo 3, Norway*

Received 17 July 1980 Accepted 10 November 1980

**Summary.—Populations of NHIK 3025 cells synchronized by mitotic selection were exposed at 37°C to extreme hypoxia in absence and presence of misonidazole (MISO). Cells in G1, S or G2 and mitosis were treated for 3 h. Inhibition of cell-cycle progression by this treatment was measured by flow cytometry of DNA histograms, and cell inactivation was measured by colony formation. The exposure to hypoxia alone of cells in G1 or in G2 and mitosis led to only minor cell-cycle inhibition, and hardly reduced cell survival. However, the exposure of cells in S to hypoxia alone had a strong inhibitory effect on cell-cycle progression, and cell survival was only 40% of untreated cells. Low concentrations of MISO (0·05–0·4 mM) during exposure of cells in S to hypoxia, produced less cell-cycle inhibition than after hypoxia alone, and cell survival was restored to 100%. The presence of MISO during the 3h exposure to hypoxia of cells in G1 or in G2 and mitosis only *increased* the effects of hypoxia alone. MISO at concentrations >0·8 mM during hypoxia produced cell inactivation, for all phases of the cell cycle, comparable to that already known from the literature.**

MISONIDAZOLE (formerly denoted Ro-07-0582, here abbreviated to MISO) not only sensitizes hypoxic cells to ionizing radiation, but by itself leads to cell inactivation. This inactivation is more pronounced under hypoxic than under aerobic conditions (Hall & Roizin-Towle, 1975; Moore *et al.*, 1976; Stratford & Adams, 1977; Denekamp, 1978). It has been shown that the stronger inactivation of hypoxic cells is caused by metabolites of MISO produced by hypoxic cells (Olive & Durand, 1978; Whitmore *et al.*, 1978) which are not produced by aerobic cells incubated with MISO. Thus, if aerobic cells are incubated with medium conditioned under hypoxia by cells in the presence of MISO, greater cell inactivation is found than after the same exposure of aerobic cells directly to MISO.

We have previously reported cell-cycle inhibition exerted by MISO on human NHIK 3025 cells under aerobic conditions (Lindmo *et al.*, 1979). The results showed that MISO induces cell-cycle inhibition in aerobic cells, even at concentrations below 1 mM. This inhibition is characterized by a reduced rate of cell-cycle progression, but only in those cells that were exposed during mitosis and/or early G1. Under hypoxic conditions more severe inhibition of the cell-cycle progression might be expected, assuming that cell inactivation and cell-cycle inhibition are only different manifestations of the same primary cell damage caused by MISO.

Presently, we have studied the effects of MISO under extreme hypoxia on the cell-cycle progression and cell survival of NHIK 3025 cells.

### MATERIALS AND METHODS

Human cells of the established line NHIK 3025 (Nordbye & Oftebro, 1969; Oftebro & Nordbye, 1969) were cultured in medium E2a (Puck *et al.*, 1957) supplemented with

† To whom correspondence should be addressed.

30% serum. Populations with a high degree of synchrony were obtained by mitotic shake-off selection from exponentially growing populations as described previously (Pettersen *et al.*, 1977). Typical values of the synchronization index as defined by Engelberg (Bakke & Pettersen, 1976) were 95% for the first and 64% for the second division after selection (Pettersen *et al.*, 1977). Under growth conditions as used here, the NHIK 3025 cells have median cell-cycle time of ~18 h, with median G1 and S durations of ~7 and ~8 h respectively. Thus, DNA synthesis starts at ~7 h and lasts until 15 h after selection (Pettersen *et al.*, 1977; Lindmo & Pettersen, 1979).

Usually mitotic selection was repeated several times at 45min intervals to provide enough cells for one experiment. For cell-kinetic studies, the yield of cells from each selection ($3\text{–}6 \times 10^5$ cells in 180 ml medium) was seeded in 6 glass Petri dishes (7 cm in diameter) which were placed in a 37°C incubator supplying automatically an atmosphere of 5% $CO_2$ in air of high humidity.

The exposure to hypoxia alone or in combination with MISO (the drug was kindly supplied by Roche Products Ltd, U.K.) was performed as follows. At the appropriate time after selection, dishes from one mitotic selection were brought from the $CO_2$ incubator into a walk-in incubator room at 37°C. The medium was removed, and for cells which were to be exposed to hypoxia alone, each dish was rinsed and filled with 3 ml medium. Each of the other dishes was rinsed with medium containing MISO at the proper concentration and then 3 ml of this medium was added. The dishes were then placed without covers in a stainless steel chamber and flushed with $N_2$ containing 3% $CO_2$ and less than 4 pts/$10^6$ $O_2$, using a set-up described in previous reports (Pettersen *et al.*, 1973; Løvhaug *et al.*, 1977). The gas mixture was humidified in a sealed water bath at 37°C with separate temperature control before entering the chamber in order to prevent evaporation of medium from the dishes. The thin layer of medium permitted rapid gas exchange, and the concentration of $O_2$ at the outflow from the chamber decreased to <4 pts/$10^6$ after about 15 min of flushing. The cells were flushed at 37°C for 3 h. The medium was then replaced by 10 ml MISO-free, well-oxygenated medium in all the dishes, and the dishes were again placed in the $CO_2$ incubator. Untreated control populations were kept in the $CO_2$ incubator all the time after mitotic selection. In some experiments the medium was changed also on the untreated control populations at the times corresponding to the beginning and end of exposure, but no effect of this medium change was found on the cell-cycle traverse.

At different times after mitotic selection, samples were trypsinized and stained for analysis of cell-cycle progression by flow cytometric measurement of DNA histograms. The cell samples were stained with mithramycin (Mithracin, Pfizer Inc., U.S.A.) without previous fixation (Crissman & Tobey, 1974) as described earlier (Lindmo & Pettersen, 1979; Lindmo *et al.*, 1979). DNA histograms were recorded on a laboratory-built flow cytometer (Lindmo & Steen, 1977) using the 457·9nm line of an Argon laser for excitation of mithramycin fluorescence (Lindmo & Pettersen, 1979). A pre-set number of cells was measured for each sample, and the histogram data were analysed to determine the fraction of cells in G1, S and G2+M (M for mitosis).

For cell-survival studies, cells from one mitotic selection were seeded in 4·5cm glass Petri dishes, using volumes of cell suspension expected to produce about 100 colonies per dish. Exposure to hypoxia alone or in combination with MISO followed the procedure explained above for cell-kinetic studies, with the exception that for these small dishes the change of medium prior to hypoxic incubation was performed using a correspondingly smaller volume of medium per dish (1·0 ml). After completion of the exposure and change of medium, the dishes were placed in the $CO_2$ incubator for 11–14 days with a change of medium on Days 5 or 6. The surviving fraction was determined by macroscopic counting of the number of colonies per dish after fixation and staining (Pettersen *et al.*, 1973).

### RESULTS

### *Exposure of cells in S*

Fig. 1 shows DNA histograms of 3 differently treated populations of synchronized cells, all trypsinized and stained 22 h after mitotic selection. The histograms have been analysed by a mathematical model (Dean & Jett, 1974; Lindmo & Aarnaes, 1979) to determine the

FIG. 1.—DNA histograms of synchronized NHIK 3025 cells trypsinized and stained at 22 h after selection. Treatment was as follows. A: Untreated control kept under aerobic conditions. B: Cells exposed to extreme hypoxia for 3 h during S, starting 10 h after mitotic selection. C: Cells treated as in B, but 0·2mM MISO present during the hypoxia. Except for the 3 h interval, the cells in B and C were kept under aerobic conditions. The full line is the curve obtained after fitting a mathematical model to the data (triangles). The broken line is the distribution of cells in S as represented in the model by a broadened exponential function. Each histogram represents 15,000 cells.

fractions of cells in G1, S and G2+M. In untreated populations (A), >80% of the cells were found in G1 at 22 h, indicating that at this time nearly all cells of the first generation after mitotic selection had completed cell division. This is in agreement with earlier reports on cell-cycle kinetics of NHIK 3025 cells (Pettersen *et al.*, 1977; Lindmo & Pettersen, 1979) which demonstrate that these cells have a median cell-cycle time of ~18 h and that ~80% of the cells divide within the interval 16–21 h after selection. The histograms in the 2 lower panels of Fig. 1 refer to populations which during the interval 10–13 h after selection were exposed to extreme hypoxia alone (B) and to hypoxia in presence of 0·2mM MISO (C). More than 90% of the cells in the population exposed to hypoxia alone were found to be in G2+M, and hardly any G1 cells were seen. Thus, cell division had not yet started in this population 22 h after mitotic selection. In the population treated with 0·2mM MISO during the period of hypoxia, ~20% were found in G1. Since ~90% of the cells had entered S at the start of exposure 10 h after selection (see below), most G1 cells seen in Fig. 1C are G1 cells of the second generation after selection. Thus cell division has started in this population at 22 h, indicating that the cell-cycle inhibition induced by hypoxia is significantly less when 0·2mM MISO is present.

To investigate the cell-cycle progression as a function of time after treatment of cells in S with hypoxia in absence and in presence of MISO, treated cell populations and untreated controls were trypsinized and stained for DNA measurement at different times up to 31 h after mitotic selection. Fig. 1 shows examples of DNA histograms from such an experiment, and data from the complete time-series of histograms from that experiment are shown in Fig. 2A. The fraction of cells in G1, as calculated from the DNA histograms, is plotted against time to serve as a measure of cell-cycle progression.

The broken lines drawn in Fig. 2 up to 14 h represent measurements from earlier control experiments, both published (Lindmo & Pettersen, 1979; Rønning *et al.*, 1980) and unpublished. These data show that about 10% of the cells are still in G1 at the start of exposure 10 h after

FIG. 2.—Fraction of NHIK 3025 cells in Gl as a function of the time after synchronization by mitotic selection. The Gl fraction was determined from the mathematical model fitted to the DNA histograms as shown by examples in Fig. 1. The symbols represent: (○) Untreated control. (△) Cells exposed to extreme hypoxia for 3 h during S, starting 10 h after selection. (◇) Cells exposed to same extreme hypoxia in presence of 0·2mM MISO (A) or 0·4 mM MISO (B). The period of treatment is indicated by the horizontal bar. The broken curve before 14 h represents means from earlier control experiments.

FIG. 3.—Fraction of NHIK 3025 cells in Gl of the 2nd generation 22 h after synchronization. Data are shown as a function of the concentration of MISO during the extreme hypoxia for 3 h during S, starting 10 h after selection. The fraction of Gl cells in the untreated control (●) is shown for reference. For clarity the ordinate scale has been displaced to the left of the origin. The number of independent observations is indicated to the right of each symbol. The difference between two observations or 1 s.e. is indicated by vertical bars if the deviation exceeds the size of the symbol.

mitotic selection. During the exposure to hypoxia (with or without MISO) the cells hardly proceed through the cell cycle (DNA histograms not shown). Therefore, the fraction of cells that have left S at the end of exposure will be insignificant, and we conclude that during the whole treatment period at least 90% of the cells were in S.

The curves in Fig. 2 demonstrate that extreme hypoxia (no MISO) from 10–13 h after selection has significantly reduced the rate of cell-cycle progression. Thus, the appearance of the first G1 cells of the second generation is delayed 8–9 h. However, if 0·2mM MISO is present during the extreme hypoxia, the delay is only about half as long (panel A).

Also, in the presence of 0·4mM MISO during hypoxia, the delay in the onset of cell division is shorter than after exposure to hypoxia alone (panel B). However, in this case the cells divide at a slower rate than after hypoxia alone, and a significant fraction of cells in S of the second generation could be seen in the DNA histogram of the population treated with hypoxia alone before this occurred in the population also exposed to 0·4mM MISO.

As seen from Fig. 2, the G1 fraction 22 h after selection represents a reasonable measure for comparing cell-cycle inhibition in the range of MISO concentrations where MISO counteracts the inhibition due to hypoxia alone. A dose-response curve based on this measure is shown in Fig. 3. The G1 fraction at 22 h for untreated cells is shown for reference. The difference in Fig. 3 between the G1 fraction of untreated cells and cells treated with hypoxia in absence of MISO, reflects the inhibition of cell-cycle progression by hypoxia alone. Fig. 3 illustrates that the effect of MISO during the treatment with extreme hypoxia is dual. At small concentrations (0·05 and 0·1 mM) a considerable increase in the G1 fraction at 22 h is seen compared to the sample with extreme hypoxia alone. This shows that such concentrations of MISO reduces the inhibition of cell-cycle progression by hypoxia in NHIK 3025 cells in S. However, as the concentration of MISO rises above 0·1 mM, the G1 fraction decreases. This suggests that MISO at such concentrations not only reduces the cell-cycle inhibition by hypoxia, but also itself induces cell-cycle inhibition, which increases with the concentration of MISO. At 0·8 mM these opposing effects of MISO produce the same delay of cell-cycle progression as hypoxia alone.

FIG. 4.—Surviving fraction of synchronized NHIK 3025 cells measured by colony-forming ability and shown as a function of the concentration of MISO during extreme hypoxia for 3 h during S, starting 10 h after selection. Surviving fraction in untreated control populations is taken at 100%. The number of independent observations and error limits are shown as in Fig. 3. The abscissa in this double-logarithmic plot is broken in order to display also the surviving fraction without MISO (hypoxia alone).

Fig. 4 shows the fraction of surviving cells as a function of the concentration of MISO during the 3h period of hypoxia starting 10 h after mitotic selection. The cell populations were treated identically to those referred to in Figs 1–3, but instead of preparing samples for flow cytometry, the cells were incubated until colonies were formed. The surviving fraction is expressed as the number of colonies in the treated population relative to the number of colonies in the untreated control. Fig. 4 shows that only about 40% of cells survive the 3h hypoxia starting 10 h after selection. However, when MISO is present in concentrations of 0·1 and 0·2 mM during the hypoxia, the surviving fraction is not significantly different from 100%. At 1·6 and 4·0mM MISO, the surviving fraction is lower than that for cells treated with hypoxia without MISO.

*Exposure of other phases of the cell cycle*

To compare the effects after exposure of cells in G to 3h hypoxia alone or with MISO with the effects seen after a similar treatment of cells in S, a 3h treatment starting 2 h after mitotic selection was chosen.

Fig. 5 illustrates the cell-cycle progression of cells treated in G1 with hypoxia alone or with 0·4mM MISO. The data were obtained from a time series of DNA histograms analysed to determine the fraction of cells in G1 as a function of time after mitotic selection. Half the cells of the untreated control had left G1 about 7 h after selection, whereas cells exposed to 3 h hypoxia were delayed ~ 2 h in reaching that level. The presence of 0·4mM MISO during hypoxia further increased this delay to 3 h.

To investigate the effects of various concentrations of MISO during hypoxia, a reasonable measure of cell-cycle progression was found to be the G1 fraction determined from DNA histograms 14 h after mitotic selection (see Fig. 5). This corresponds to 9 h after the end of the treatment, which was also selected for similar analysis after treatment of cells in

FIG. 5.—Fraction of NHIK 3025 cells in G1 as a function of time after synchronization. The G1 fraction was determined from DNA histograms by a planimetric procedure. The symbols represent: (○) Untreated control. (△) Cells in extreme hypoxia for 3 h during G1, starting at 2 h after selection. (◇) Cells exposed to the same extreme hypoxia in presence of 0·4mM MISO. The period of treatment is indicated by the horizontal bar. The broken curve is explained in the legend to Fig. 2.

FIG. 6.—Fraction of NHIK 3025 cells remaining in G1 14 h after synchronization, shown as a function of the concentration of MISO present during extreme hypoxia for 3 h during G1, starting 2 h after selection. The number of independent observations and error limits are indicated as in Fig. 3. The fraction of G1 cells in the untreated control 14 h after selection (●) is shown for reference. For clarity the ordinate scale has been displaced to the left of the origin.

S, thus facilitating a comparison between the results obtained for the two stages of the cell cycle. A dose–response curve based on this measure is shown in Fig. 6, where the G1 fraction of the untreated control population is also shown for comparison. Fig. 6 demonstrates that exposure of cells in G1 to hypoxia alone leads to only minor inhibition of cell-cycle progression compared to the effect of exposure of cells in S (Fig. 3). Furthermore, after exposure of cells in G1, inhibition of cell-cycle progression continuously increases with increasing concentrations of MISO during hypoxia, thus demonstrating a fundamentally different relationship from that found after treatment of cells in S (Fig. 3).

Fig. 7 (closed symbols) shows the cell survival after the 3h exposure of G1 cells to hypoxia with various concentrations of MISO. Like the data on cell-cycle inhibition (Fig. 6) hypoxia alone has only a minor effect, and no significant reduction of this effect can be demonstrated for any concentration of MISO.

In an attempt to induce a greater effect by hypoxia alone, G1 cells were exposed to extreme hypoxia for 12 h (starting 2 h after mitotic selection). DNA histograms showed that the cells remained in G1 during the whole treatment. As Fig. 7 demonstrates (open symbols), cell inactivation by hypoxia alone was not significantly larger than after the usual 3h treatment. However, the concentration of MISO required to induce a given inactivation in the prolonged treatment was only about 1/10 of that required in the 3h treatment.

Specific exposure of cells in G2+M was difficult to achieve, since the degree of synchrony deteriorates with time after mitotic selection, and since the desired 3h exposure is nearly as long as the median duration of G2+M. The results of an experiment in which the cells were exposed to hypoxia alone and in presence of 0·4mM MISO for 3 h, starting 15 h after

FIG. 7.—Surviving fraction of synchronized NHIK 3025 cells measured by colony-forming ability and shown as a function of the concentration of MISO during extreme hypoxia. Exposure started 2 h after mitotic selection and lasted for 3 h (closed symbols) or 12 h (open symbols). Values are shown relative to the surviving fraction in control populations (taken at 100%). The number of independent observations and error limits are indicated as in Fig. 3. The abscissa in this double-logarithmic plot is broken to display the surviving fraction without MISO (hypoxia alone).

FIG. 8.—Fraction of NHIK 3025 cells in G1 as a function of the time after synchronization. The G1 fraction was determined from DNA histograms by fitting a mathematical model. The symbols represent: (○) Untreated control. (△) Extreme hypoxia for 3 h during G2, M and early G1, starting 15 h after selection. (◇) Extreme hypoxia as above, with 0·4mM MISO. The period of treatment is indicated by the horizontal bar. The broken curve is as in Fig. 2.

mitotic selection, is shown in Fig. 8. About 10% of the cells had already divided when treatment was started, and during treatment cell division went on relatively undisturbed. The DNA histograms showed that, in the population treated with hypoxia alone, the entrance of cells into S of the second generation started at about the same time (22 h after selection) as for untreated cells, but the cells proceeded at a somewhat lower rate. Cell-cycle progression in this second generation was, however, severely inhibited in the cells exposed to 0·4mM MISO during hypoxia, and in the DNA histogram no significant entry of cells into S was seen until 32 h after mitotic selection (*i.e.* 10 h later than in the untreated control and in the population exposed to hypoxia alone).

Neither hypoxia alone nor additional MISO up to 0·4 mM caused any significant cell inactivation (survival 80–90%) in an experiment performed with a 3h treatment starting 15 h after mitotic selection (results not shown).

### DISCUSSION

### *Effects of extreme hypoxia*

The results in Figs 1, 2, 5 and 8 demonstrate that 3h exposure to extreme hypoxia during G1, S or G2+M inhibits cell-cycle progression. Whereas cell division could take place during hypoxia (Fig. 8) cells exposed to hypoxia in S hardly proceeded through the cell cycle during treatment. Cells treated in G1 seemed to proceed through G1 but would not enter S under hypoxia. This is shown by DNA histograms of exponentially growing NHIK 3025 cells treated with extreme hypoxia (Fig. 9). The accumulation of cells in late G1 during hypoxia led to a synchronous entry into S shortly after reoxygenation (panel C). Three h after reoxygenation these cells had achieved DNA synthesis corresponding to about 2h synthesis at the normal rate.

The cell-cycle progression of synchronized cells was slowed also after the end of the treatment, thus indicating lasting effects of the damage caused by hypoxia.

FIG. 9.—DNA histograms of exponentially growing NHIK 3025 cells treated with extreme hypoxia. A: At start of treatment. Aerobic control populations show a stable cell-cycle distribution with 50 ± 2% in G1, 34 ± 1% in S and 16 ± 2% in G2+M. B: End of 12 h hypoxia. 62% G1, 28% S and 10% G2 + M, suggesting that half the population of G2 + M cells have divided and that 10% of S have entered G2. C: 3 h after reoxygenation. The fraction of cells in S is 60–65%.

The inhibition was strongest in cells exposed during S, in good agreement with the cell inactivation caused by hypoxia which also was more severe in cells in S (Figs 4 and 7). Since 12h extreme hypoxia produced in cells in G1 only the same minor cell inactivation as the 3h treatment (Fig. 7), whereas the latter treatment of cells in S induced a pronounced effect, NHIK 3025 cells demonstrate a cell-cycle phase-specific sensitivity to extreme hypoxia.

Earlier reports mostly present data on survival of asynchronous cell populations exposed to hypoxia for different lengths of time (Littbrand & Révész, 1968; Bedford & Mitchell, 1974; Born *et al.*, 1976) but the effect of prolonged hypoxia on cell kinetics has also been studied (Born *et al.*, 1976). The latter work demonstrated prolongation of G1 and S up to several times their normal duration in Chinese hamster cells, whereas the duration of G2 remained unchanged; which supports the present findings. Data on the effect of hypoxia on cell survival, as summarized by Born *et al.* (1976), demonstrate a considerable spread and variation in shape of the curves showing cell-survival as a function of the duration of hypoxia. NHIK 3025 cells, being resistant to hypoxia in G1 (and probably in G2 and M) but sensitive in S, may give rise to a biphasic cell-survival curve as a function of the duration of hypoxia.

The high sensitivity to hypoxia in S may be related to a strong requirement for $O_2$. Robbins & Morrill (1969) found a close correlation between DNA synthesis and $O_2$ uptake through the cell cycle in synchronized HeLa cells. It would seem possible that cells in S, due to an increased $O_2$ requirement, could deplete the medium of $O_2$ more efficiently than cells in other parts of the cell cycle, and thus by themselves aggravate the degree of hypoxia. Consequently one would expect to find more severe effects of hypoxia on cells in S. This possibility does, however, seem remote with the present experimental procedure using very low cell densities (10–30 cells/cm$^2$ in survival studies), thin layer of medium (< 1 mm during hypoxia) and constant flushing (5 l/min) of the stainless-steel incubation chamber with gas of fixed composition (< 4 pts/$10^6$ $O_2$ in $N_2$, with 3% $CO_2$).

High $O_2$ requirement during S may, however, explain why hypoxia arrests the cells in a pre-DNA-synthetic stage, as demonstrated in Fig. 9. Similar observations have been made by others (Koch *et al.*, 1973; Bedford & Mitchell, 1974) and increased fractions of G0/G1 cells have been found in the inner, hypoxic region of multicellular spheroids (Lücke-Huhle & Dertinger, 1977), an observation also registered for NHIK 3025 cells (Wibe *et al.*, 1981).

*Effects of MISO during extreme hypoxia*

Fig. 2 shows that while cells exposed in S to extreme hypoxia alone are delayed in entering G1 of the next generation by about 9 h, extreme hypoxia with 0·2mM MISO induces only half that delay. Lower concentrations of MISO (0·05 and 0·1 mM) are even more efficient than 0·2 mM in reducing the cell-cycle inhibition caused by hypoxia alone (Fig. 3). Thus, MISO counteracts the cell-cycle inhibition by extreme hypoxia alone on cells in S.

This protective effect is not limited to inhibition of the cell cycle. Fig. 4 shows a protective effect also on the colony-forming ability after treatment of cells in S. Whilst survival after hypoxia alone is about 40%, lethal damage is avoided when MISO in concentrations of 0·1–0·2 mM is present during hypoxia, and cell survival is restored to control values. The cell inactivation at higher concentrations of MISO is probably due to its metabolites known to be formed under hypoxic conditions (Olive & Durand, 1978; Whitmore *et al.*, 1978).

Presence of MISO during exposure of cells in G1 or G2+M to hypoxia leads to only additional cell-cycle inhibition and cell inactivation (Figs 5, 6, 7 and 8).

By comparing the effects of hypoxia on cells in G1, S or G2+M in presence of 0·4mM MISO (Figs 2B, 5 and 8) it is notable that the additional cell-cycle inhibition by 0·4mM MISO when cells are exposed during division is more pronounced than after exposure at other stages of the cell cycle. This accords with our previous studies on cell-cycle inhibition by MISO under aerobic conditions (Lindmo *et al.*, 1979) which demonstrated that MISO under aerobic conditions reduces the rate of cell-cycle progression only in cells exposed during mitosis/early G1.

Cell inactivation due to concentrations of MISO above 1 mM are comparable to results obtained by other workers (Hall & Roizin-Towle, 1975; Moore *et al.*, 1976; Born *et al.*, 1976; Stratford & Adams, 1977; Hall *et al.*, 1977; Stratford & Gray, 1978; Taylor & Rauth, 1978; Geard *et al.*, 1978; Wong *et al.*, 1978; Hall *et al.*, 1979; Astor & Hall, 1979; Stratford *et al.*, 1980; Taylor & Rauth, 1980). Surviving fractions after 3h exposure of exponentially growing cells to hypoxia and 5mM MISO range from 0·1–60% for V79 cells (Stratford & Adams, 1977; Geard *et al.*, 1978), from 0·6–35% for CHO cells (Wong *et al.*, 1978; Taylor & Rauth, 1980), but were as low as $10^{-4}$–$10^{-5}$ for HeLa cells (Taylor & Rauth, 1978; 1980).

NHIK 3025 cells seem to present a large difference in sensitivity between G1 and S. Whereas Hall & Biaglow (1977) concluded that in synchronized CHO cells, cell inactivation by 5mM MISO for 3 h under hypoxia is almost independent of the position in the cell cycle, our results (Figs 4 and 7) suggest a 20× greater survival of G1 cells than S cells after a similar exposure. The survival of NHIK 3025 cells after exposure for different times in G1 and S to extreme hypoxia and 30mM MISO at room temperature has previously been studied (Pettersen, 1978). After a 3h exposure the surviving fraction of cells in G1 was about 30× that of cells in S.

The present study demonstrates a protective effect of MISO on cells in S exposed to hypoxia, which reaches its maximum (*i.e.* 100% survival) already at the low MISO concentration of 0·1 mM. Since hypoxic cells metabolize MISO, the concentration of MISO will decrease, whereas the concentration of metabolites will increase with increasing duration of hypoxia. The observed protection may therefore vary with treatment time, depending on how efficient the metabolites of MISO are in exerting this protection, compared to MISO itself.

It is known that hypoxic-cell sensitizers influence cellular $O_2$ use (Biaglow, 1980). MISO in high concentrations (5 mM) has thus been found to inhibit $O_2$ utilization in aerobic V79 cells by 20% (Biaglow *et al.*, 1978; Durand *et al.*, 1978). The strong cell-cycle inhibition and cell inactivation after exposure of NHIK 3025 cells in S to extreme hypoxia alone, might be due to an aggravation of the hypoxia by cellular $O_2$ utilization. If so, the presence of MISO would seem to counteract the effects of hypoxia alone by preventing this $O_2$ utilization. MISO is, however, one of the radiosensitizing nitrocompounds with the least capacity for inhibiting cellular $O_2$ utilization. In fact, 1mM MISO was found to have no effect on the rate of $O_2$ consumption in Ehrlich tumour cells or V79 lung cells (Biaglow *et al.*, 1978; Durand *et al.*, 1978) and even stimulation of cellular $O_2$ consumption by 10mM MISO has been found in Ehrlich ascites cells (Mustea *et al.*, 1978). The possibility that MISO in concentrations of 0·1 mM should significantly reduce $O_2$ utilization in NHIK 3025 cells under extreme hypoxia therefore seems remote.

Nevertheless, an attempt was made to clarify whether the severe effects of hypoxia on cells in S were due to an aggravation of hypoxia caused by cellular utilization of residual $O_2$. Synchronized NHIK 3025 cells in S were subjected for 3 h to extreme hypoxia, but in presence of various concentrations of rotenone instead of MISO. Rotenone in concentrations about 0·1–1 $\mu$M is routinely used to inhibit cellular respiration (Gregg *et al.*, 1968; Durand & Biaglow, 1977; Biaglow *et al.*, 1978). We used concentrations in the range 0·3–10 $\mu$M which caused no cell inactivation under aerobic conditions. Rotenone led to no increased survival of cells in S exposed to extreme hypoxia. The way in which MISO protects cells in S exposed to hypoxia therefore remains unexplained.

Some mechanisms behind the effects of MISO as a radiosensitizer may be of interest in relation to the observed protective effect. The direct-action model for radiosensitization by electron-affinic chemicals (Adams & Cooke, 1969) assumes the polarization of a target molecule as the primary radiation damage. The sensitizer presumably acts by influencing the competition between two different reactions in the polarized target molecule: (i) repair of the target molecule by charge recombination, or (ii) fixation of the damage by transfer of an electron from the target molecule to the sensitizer. $O_2$ and other electron-affinic chemicals such as MISO belong by this model to the same group of radiosensitizers.

The high electron-affinity of $O_2$, which according to the above model determines its properties as a radiosensitizer, is also fundamental for its role in respiration, where $O_2$ acts as an acceptor of electrons in the electron transport system. An electron-affinic chemical like MISO, able to mimic the radiosensitizing effects of $O_2$, might therefore also be able to mimic the respiratory effect of $O_2$.

If the protective effect of MISO on cells in S is a general phenomenon, demonstrable also in other cell types and *in vivo*, it may be of clinical interest to note that the full effect requires only a very low concentration of MISO. In clinical use of MISO as a radiosensitizer, peak concentrations of about 0·5 mM in plasma as well as tumour tissue are readily attainable (Gray *et al.*, 1976; Ash *et al.*, 1979). Considering the half life of MISO in humans (Dische *et al.*, 1978) it seems that hypoxic cells in the tumour will be exposed for several days to MISO concentrations high enough for the full protection as seen here on NHIK 3025 cells in S. The time dependence of the effect will therefore be important, and the question discussed above; whether or not the metabolites of MISO formed under hypoxia are able to counteract the cell-cycle inhibition and cell inactivation induced by hypoxia alone, becomes essential. Data which show that some of these metabolites are equally efficient radiosensitizers to MISO (Brown *et al.*, 1979; Flockhart *et al.*, 1978) are interesting in this respect.

The authors gratefully acknowledge the assistance of Kåre Fundingsrud who skilfully operated the flow cytometer. This work was supported by the Norwegian Cancer Society—Landsforeningen mot Kreft.

### REFERENCES

ADAMS, G. E. & COOKE, M. S. (1969) Electron-affinic sensitization. I. A structural basis for chemical radiosensitizers in bacteria. *Int. J. Radiat. Biol.*, **15**, 457.

ASH, D. V., SMITH, M. R. & BUGDEN, R. D. (1979) Distribution of misonidazole in human tumours and normal tissues. *Br. J. Cancer*, **39**, 503.

ASTOR, M. & HALL, E. J. (1979) Misonidazole and MTDQ in combination: Cytotoxic and radio-sensitizing properties in hypoxic mammalian cells. *Br. J. Cancer*, **39**, 510.

BAKKE, O. & PETTERSEN, E. O. (1976) A fast and accurate method for calculating Engelberg's synchronization index. *Cell Tissue Kinet.*, **9**, 389.

BEDFORD, J. S. & MITCHELL, J. B. (1974) The effect of hypoxia on the growth and radiation response of mammalian cells in culture. *Br. J. Radiol.*, **47**, 687.

BIAGLOW, J. E. (1980) The effect of hypoxic cell radiosensitizing drugs on cellular oxygen utilization. *Pharmacol. Ther.*, **10**, 283.

BIAGLOW, J. E., GREENSTOCK, C. L. & DURAND, R. E. (1978) Effects of sensitizers on cell respiration: I. Factors influencing the effects of hypoxic cell radiosensitizers on oxygen utilization of tumour and cultured mammalian cells. *Br. J. Cancer*, **37**, Suppl. III, 145.

BORN, R., HUG, O. & TROTT, K. R. (1976) The effect of prolonged hypoxia on growth and viability of Chinese hamster cells. *Int. J. Radiat. Oncol. Biol. Phys.*, **1**, 687.

BROWN, J. M., YU, N. Y. & WORKMAN, P. (1979) Pharmacokinetic considerations in testing hypoxic cell radiosensitizers in mouse tumours. *Br. J. Cancer*, **39**, 310.

CRISSMAN, H. A. & TOBEY, R. A. (1974) Cell-cycle analysis in 20 minutes. *Science*, **184**, 1297.

DEAN, P. N. & JETT, J. H. (1974) Mathematical analysis of DNA distributions derived from flow microfluorometry. *J. Cell Biol.*, **60**, 523.

DENEKAMP, J. (1978) Cytotoxicity and radiosensitization in mouse and man. *Br. J. Radiol.*, **51**, 636.

DISCHE, S., SAUNDERS, M. I. & FLOCKHART, I. R. (1978) The optimum regime for the administration of misonidazole and the establishment of multi-centre clinical trials. *Br. J. Cancer*, **37**, Suppl. III, 318.

DURAND, R. E. & BIAGLOW, J. E. (1977) Radio-sensitization of hypoxic cells of an *in vitro* tumor model by respiration inhibitors. *Radiat. Res.*, **69**, 359.

DURAND, R. E., BIAGLOW, J. E. & GREENSTOCK, C. L. (1978) Effects of sensitizers on cell respiration: III. The effects of hypoxic cell radiosensitizers on oxidative metabolism and the radiation response of an *in vitro* tumour model. *Br. J. Cancer*, **37**, Suppl. III, 150.

FLOCKHART, I. R., SHELDON, P. W., STRATFORD, I. J. & WATTS, M. E. (1978) A metabolite of the 2-nitroimidazole misonidazole with radio-sensitizing properties. *Int. J. Radiat. Biol.*, **34**, 91.

GEARD, C. R., POVLAS, S. F., ASTOR, M. B. & HALL, E. J. (1978) Cytological effects of 1-(2-nitro-1-imidazolyl)-3-methoxy-2-propanol (misonidazole) on hypoxic mammalian cells *in vitro*. *Cancer Res.*, **38**, 644.

GRAY, A. J., DISCHE, S., ADAMS, G. E., FLOCKHART, I. R. & FOSTER, J. L. (1976) Clinical testing of the radiosensitizer Ro-07-0582. I. Dose tolerance, serum and tumour concentrations. *Clin. Radiol.*, **27**, 151.

GREGG, C. T., MACHINIST, J. M. & CURRIE, W. D. (1968) Glycolytic and respiratory properties of intact mammalian cells: Inhibitor studies. *Arch. Biochem. Biophys.*, **127**, 101.

HALL, E. J., ASTOR, M., OSMAK, R., SHAPIRO, P. & AUGUST, L. (1979) A comparison of two nitro-imidazoles and a dihydroquinoline as radio-sensitizers and cytotoxic agents. *Int. J. Radiat. Oncol. Biol. Phys.*, **5**, 1781.

HALL, E. J., ASTOR, M., GEARD, C. & BIAGLOW, J. (1977) Cytotoxicity of Ro-07-0582; enhancement by hyperthermia and protection by cysteamine. *Br. J. Cancer*, **35**, 809.

HALL, E. J. & BIAGLOW, J. (1977) Ro-07-0582 as a radiosensitizer and cytotoxic agent. *Int. J. Radiat. Oncol. Biol. Phys.*, **2**, 521.

HALL, E. J. & ROIZIN-TOWLE, L. (1975) Hypoxic sensitizers: Radiobiological studies at the cellular level. *Radiology*, **117**, 453.

KOCH, C. J., KRUUV, J., FREY, H. E. & SNYDER, R. A. (1973) Plateau phase in growth induced by hypoxia. *Int. J. Radiat. Biol.*, **23**, 67.

LINDMO, T. & AARNAES, E. (1979) Selection of optimal model for the DNA histogram by analysis of error of estimated parameters. *J. Histochem. Cytochem.*, **27**, 297.

LINDMO, T. & PETTERSEN, E. O. (1979) Delay of cell cycle progression after X-irradiation of synchronized populations of human cells (NHIK 3025) in culture. *Cell Tissue Kinet.*, **12**, 43.

LINDMO, T., PETTERSEN, E. O. & WIBE, E. (1979) Cell-cycle inhibition by misonidazole of human cells cultivated *in vitro* under aerobic conditions. *Br. J. Cancer*, **40**, 755.

LINDMO, T. & STEEN, H. B. (1977) Flow cytometric measurement of the polarization of fluorescence from intracellular fluorescein in mammalian cells. *Biophys. J.*, **18**, 173.

LITTBRAND, B. & RÉVÉSZ, L. (1968) Survival of cells in anoxia. *Br. J. Radiol.*, **41**, 479.

LÜCKE-HUHLE, C. & DERTINGER, H. (1977) Kinetic response of an *in vitro* "tumour model" (V79 spheroids) to 42°C hyperthermia. *Eur. J. Cancer*, **13**, 23.

LØVHAUG, D., WIBE, E., OFTEBRO, R., PETTERSEN, E. O. & BRUSTAD, T. (1977) Recovery from X-ray induced damage in human cells grown in culture. *Neoplasma*, **24**, 513.

MOORE, B. A., PALCIC, B. & SKARSGARD, L. D. (1976) Radiosensitizing and toxic effects of the 2-nitroimidazole Ro-07-0582 in hypoxic mammalian cells. *Radiat. Res.*, **67**, 459.

MUSTEA, I., BARA, A., PETRESCU, I. & RÉVÉSZ, L. (1978) Effect of anoxic radiosensitizers on cellular and mitochondrial oxygen consumption and respiration control ratio. *Br. J. Cancer*, **37**, Suppl. III, 159.

NORDBYE, K. & OFTEBRO, R. (1969) Establishment of four new cell strains from human uterine cervix. I. *Exp. Cell Res.*, **58**, 458.

OFTEBRO, R. & NORDBYE, K. (1969) Establishment of four new cell strains from human uterine cervix. II. *Exp. Cell Res.*, **58**, 459.

OLIVE, P. L. & DURAND, R. E. (1978) Activation of radiosensitizers by hypoxic cells. *Br. J. Cancer*, **37**, Suppl. III, 124.

PETTERSEN, E. O. (1978) Radiosensitizing and toxic effects of the 2-nitroimidazole Ro-07-0582 in different phases of the cell cycle of extremely hypoxic human cells *in vitro*. *Radiat. Res.*, **73**, 180.

PETTERSEN, E. O., BAKKE, O., LINDMO, T. & OFTEBRO, R. (1977) Cell cycle characteristics of synchronized and asynchronous populations of human cells and effect of cooling of selected mitotic cells. *Cell Tissue Kinet.*, **10**, 511.

PETTERSEN, E. O., OFTEBRO, R. & BRUSTAD, T. (1973) X-ray inactivation of human cells in tissue culture under aerobic and extremely hypoxic conditions in the presence and absence of TMPN. *Int. J. Radiat. Biol.*, **24**, 285.

PUCK, T. T., CIECIURA, S. J. & FISHER, H. W. (1957) Clonal growth *in vitro* of human cells with fibroblastic morphology. *J. Exp. Med.*, **106**, 145.

ROBBINS, E. & MORRILL, G. A. (1969) Oxygen uptake during the HeLa cell life cycle and its correlation with macromolecular synthesis. *J. Cell Biol.*, **43**, 629.

RØNNING, Ø. W., LINDMO, T., PETTERSEN, E. O. & SEGLEN, P. O. (1980) Kinetics of entry into S-phase and into the Gl-phase of the subsequent cell cycle for synchronized NHIK 3025 cells. In *Flow Cytometry IV*, Ed. Laerum *et al.* Oslo: Universitetsforlaget. p. 350.

STRATFORD, I. J. & ADAMS, G. E. (1977) Effects of hyperthermia on differential cytotoxicity of a hypoxic cell radiosensitizer, Ro-07-0582, on mammalian cells *in vitro*. *Br. J. Cancer*, **35**, 307.

STRATFORD, I. J. & GRAY, P. (1978) Some factors affecting the specific toxicity of misonidazole towards hypoxic mammalian cells. *Br. J. Cancer*, **37**, Suppl. III, 129.

STRATFORD, I. J., WILLIAMSON, C. & ADAMS, G. E. (1980) Combination studies with misonidazole and a cis-platinum complex: Cytotoxicity and radiosensitization *in vitro*. *Br. J. Cancer*, **41**, 517.

TAYLOR, Y. C. & RAUTH, A. M. (1978) Differences in the toxicity and metabolism of the 2-nitroimidazole misonidazole (Ro-07-0582) in HeLa and Chinese hamster ovary cells. *Cancer Res.*, **38**, 2745.

TAYLOR, Y. C. & RAUTH, A. M. (1980) Sulphydryls, ascorbate and oxygen as modifiers of the toxicity and metabolism of misonidazole *in vitro*. *Br. J. Cancer*, **41**, 892.

WHITMORE, G. F., GULYAS, S. & VARGHESE, A. J. (1978) Sensitizing and toxicity properties of misonidazole and its derivatives. *Br. J. Cancer*, **37**, Suppl. III, 115.

WIBE, E., LINDMO, T. & KAALHOS, O. (1981) Cell kinetic characteristics in different parts of multicellular spheroids of human origin. *Cell Tissue Kinet.* (In press).

WONG, T. W., WHITMORE, G. F. & GULYAS, S. (1978) Studies on the toxicity and radiosensitizing ability of misonidazole under conditions of prolonged incubation. *Radiat. Res.*, **75**, 541.